# A Fast Specular Highlight Removal Method for Smooth Liquor Bottle Surface Combined with U^2^-Net and LaMa Model

**DOI:** 10.3390/s22249834

**Published:** 2022-12-14

**Authors:** Shaojie Guo, Xiaogang Wang, Jiayi Zhou, Zewei Lian

**Affiliations:** 1 School of Automation and Information Engineering, Sichuan University of Science and Engineering, No. 188 University Road, Cuiping District, Yibin 644000, China; 2 Artificial Intelligence Key Laboratory of Sichuan Province, No. 188 University Road, Cuiping District, Yibin 644000, China

**Keywords:** smooth liquor bottle surface, small sample highlight dataset, specular highlight detection, specular highlight removal, U^2^-Net, LaMa

## Abstract

Highlight removal is a critical and challenging problem. In view of the complex highlight phenomenon on the surface of smooth liquor bottles in natural scenes, the traditional highlight removal algorithms cannot semantically disambiguate between all-white or near-white materials and highlights, and the recent highlight removal algorithms based on deep learning lack flexibility in network architecture, have network training difficulties and have insufficient object applicability. As a result, they cannot accurately locate and remove highlights in the face of some small sample highlight datasets with strong pertinence, which reduces the performance of some tasks. Therefore, this paper proposes a fast highlight removal method combining U^2^-Net and LaMa. The method consists of two stages. In the first stage, the U^2^-Net network is used to detect the specular reflection component in the liquor bottle input image and generate the mask map for the highlight area in batches. In the second stage, the liquor bottle input image and the mask map generated by the U^2^-Net are input to the LaMa network, and the surface highlights of the smooth liquor bottle are removed by relying on the powerful image inpainting performance of LaMa. Experiments on our self-made liquor bottle surface highlight dataset showed that this method outperformed other advanced methods in highlight detection and removal.

## 1. Introduction

Most of the materials in a natural scene will show a specular highlight on their surfaces under light conditions, which is particularly obvious on smooth surface materials. The removal of specular highlights has always been a key issue in computer vision and image processing.

**Figure 1:**
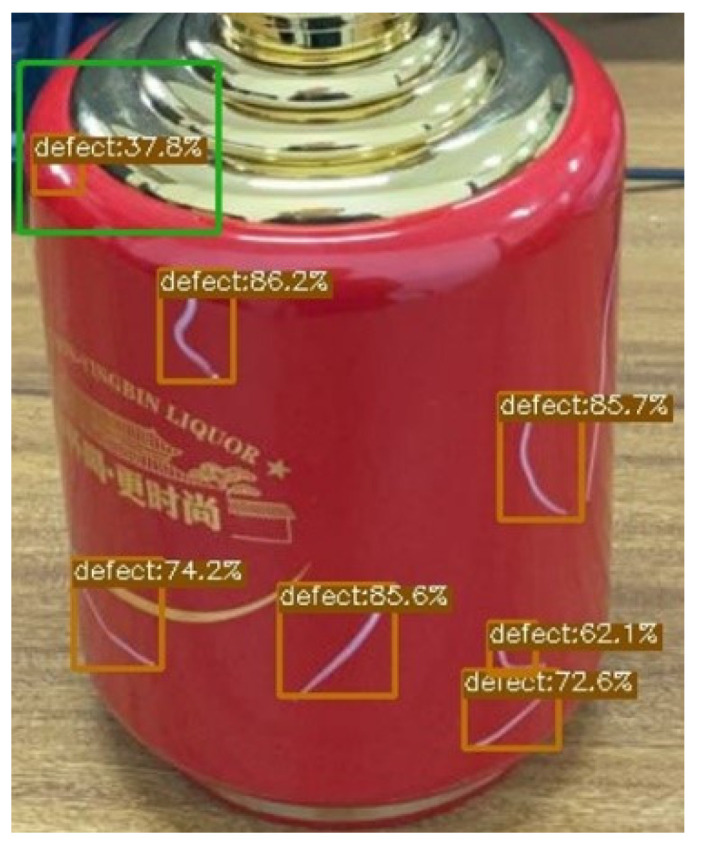
The green box is the false detection of defects caused by highlights.

In practical industrial applications, the presence of a highlight will have a serious impact on many aspects of production. The most common impact is that the presence of specular highlights will bring noise and interference to the original image, thereby reducing the performance of certain tasks such as small target detection [1,2], tracking and recognition [3]. Specifically, some automated techniques based on computer vision, such as image segmentation [4], object detection [5], tracking and recognition, all rely heavily on the color or saturation intensity information of the image itself. The presence of multiregion complex highlights can severely degrade image quality and unpredictably tamper with the raw information and intensity values of pixels, resulting in a dramatic drop in the performance of these automated techniques. For example, a highlight area similar to the target to be detected may be incorrectly identified as a target for detection; the false detection phenomenon in this real-world scenario is shown in Figure 1, and it can be seen that there is a large area of highlights on the surface of the liquor bottle, which leads to the wrong identification of white scratch defects on the surface of the liquor bottle to a certain extent.

However, in the face of some highly targeted highlight datasets, most of the existing highlight removal methods have limitations in the principle of highlight removal and the scope of application of the object, which leads to the fact that they are unable to accurately and quickly locate and remove the highlight in the true sense semantically, and the applicability is not strong.

Before introducing our proposed method, we briefly review the highlight removal techniques proposed by researchers in related fields in recent years. Generally, the most commonly used highlight removal methods are mainly divided into two categories, namely the traditional method based on the two-color reflection model [6,7,8,9,10] and the existing method based on deep learning [11,12,13].

Most of the traditional methods based on the two-color reflection model operate on various forms of threshold, and these threshold operations mainly treat the brightest pixel as a highlight. Tan et al. [14] developed a method based on chromaticity analysis, which is based only on color, especially chromaticity, and hardly needs any geometric information. Guo et al. [15] proposed a sparse and low-rank reflection model for specular highlight detection and removal using a single input image. Yang et al. [16] proposed a method based on an edge-preserving low-pass filter to smooth the maximum proportion of color components in the original image, so as to remove the highlight regarded as noise from the specular pixels. Li et al. [7] proposed an adaptive robust principal component analysis method to eliminate specular reflection in endoscopic image sequences, and it can iteratively optimize sparse part parameters during RPCA decomposition. This kind of method lacks the use of high-level semantic information. In the face of some images with bright surface colors in natural scenes and when the color information of the target to be detected is similar to the highlight, the performance is degraded, which leads to the difficulty of highlight localization, slow processing speed and poor practical value.

Compared with the above traditional methods, most of the recent methods for removing highlights are based on deep learning. Such solutions require that the algorithm can not only ‘understand‘ the large-scale structure of natural scene images, but also synthesize images when performing highlight image processing. Muhammad et al. [17] proposed Spec-Net and Spec-CGAN. Spec-Net uses intensity channels as input to remove high-intensity specular reflections from low-color images. Spec-CGAN uses RGB images as input to generate diffuse images. Hu et al. [13] further converted the highlight removal problem into image-to-image conversion by using Cycle-GAN; in their proposed network, Cycle-GAN combines a method based on non-negative matrix factorization (NMF) to obtain an accurate highlight mask to remove highlight. Fu et al. [11] first developed a specular highlight detection network (SHD-Net) based on deep learning, which uses multiscale context contrast features to accurately detect specular highlights of different scales. Then, they proposed a novel joint highlight detection and removal multitask network [18], aiming to detect and remove highlights in natural images, and achieved excellent results on their datasets.

Although the method based on deep learning improves the performance of specular highlight removal to a certain extent, for different characteristics of the dataset, the network training is not stable, resulting in a difficult training process. The reason is that these methods not only require a lot of training data and a detailed testing process, but they also need to strictly adjust the parameters of the model. Therefore, due to the limitations of the existing advanced traditional methods and some methods based on deep learning in the highlight removal principle, network training and model parameter adjustment, these methods are not effective in the face of some practical problems and cannot be well applied.

Based on the above problems, this paper proposes a simple and effective method that is based on the idea of image segmentation and inpainting and is combined with the U^2^-Net [19] and LaMa [20] network to remove the highlight on the surface of a smooth liquor bottle. The idea is inspired by a recently proposed new method called large mask inpainting (LaMa), an inpainting network that improves state-of-the-art technology for a range of datasets to achieve superior performance even in challenging scenarios. The method consists of two stages. In the first stage, the U^2^-Net network is used to detect the specular reflection component in the liquor bottle input image and generate the mask map for the highlight area in batches. In the second stage, the liquor bottle input image and the mask map generated by U^2^-Net are input to the LaMa network, and the surface highlights of the smooth liquor bottle are removed by relying on the powerful image inpainting performance of LaMa.

## 2. Analysis of Representative Problems

Most of the traditional methods based on the two-color reflection model operate on various forms of threshold, and these threshold operations mainly treat the brightest pixel as a highlight. Among them, the more advanced and representative is a high-quality pixel clustering method proposed by Antonio C.S. Souza [21]. This method first estimates the minimum and maximum chromaticity values of each pixel; then, the distribution pattern of these values in the minimum–maximum chromaticity space is analyzed to propose an effective pixel clustering method. Finally, the intensity ratio of each cluster is estimated to separate the diffuse and specular reflection components, allowing for the real-time removal of specular highlights from a single image. Compared with the general highlight removal algorithm based on the two-color reflection model, the algorithm has a better real-time performance, faster processing speed and higher processing quality.

**Figure 2:**
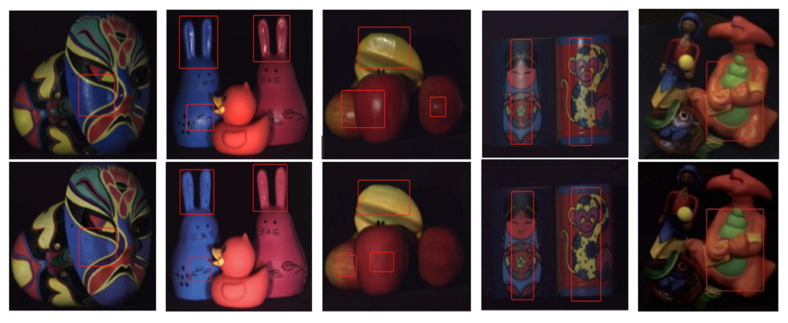
Experimental objects and results of traditional algorithm based on two-color reflection model.

**Figure 3:**
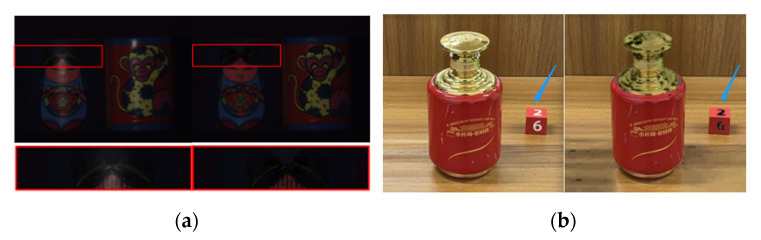
High quality pixel clustering method based on two-color reflection model: (**a**) surface color dim and generally smooth; (**b**) colorful and smooth surface.

Most of the objects handled with these traditional methods are shown in Figure 2. They all have a few things in common, that is, the overall surface is dark, the surface of the material is smooth and it does not have the characteristics of highlight objects in random real scenes. Therefore, it is difficult to locate the highlight with this kind of method, the processing speed is slow and the practical value is general when facing objects with a bright color in the real scene and when the color information of the target to be detected is similar to the highlight. Figure 3a shows the results of Antonio C.S. Souza et al. on their test dataset. It can be seen that their method can handle the highlight on the texture object well and can maintain the original brightness of the image diffuse reflection region. However, for the real image where the material surface is smooth and bright and the color information of the object to be detected is similar to the highlight, the pixel information of the highlight area separated by detection is destroyed, and the color characteristics of the original image are not preserved. Additionally, due to the inherent defects of the traditional algorithm principle, the white pixel area on the building block shown in Figure 3b is also falsely detected as a highlight. Essentially, most of the existing traditional methods cannot semantically disambiguate the ambiguity between all-white or near-white materials and highlights in complex natural real scenes, and they cannot accurately locate the real highlights.

**Figure 4:**
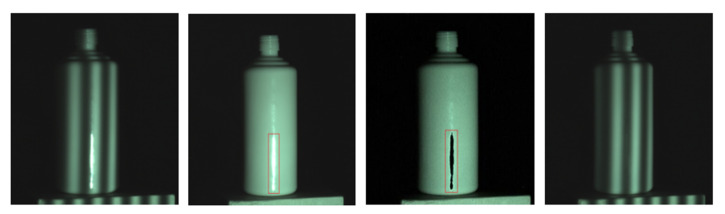
Sun et al.’s processing results of simple highlight areas on ceramic bottles.

To make up for the shortcomings of traditional algorithms in principle, Sun et al. [22] designed a new algorithm based on reflection component separation (RCS) and priority region filling theory. This method finds the specular highlight pixels in the image by comparing the pixel parameters. On this basis, the reflection components are separated and processed. However, for objects with bright and smooth surfaces that easily show strong highlights, the RCS theory will change the color information of the specular pixels due to the larger specular component. In this case, priority region filling theory is used to recover the color information, and Figure 4 shows the result of this algorithm for separating the reflection components and pixel filling the regions whose color information is changed.

**Figure 5:**
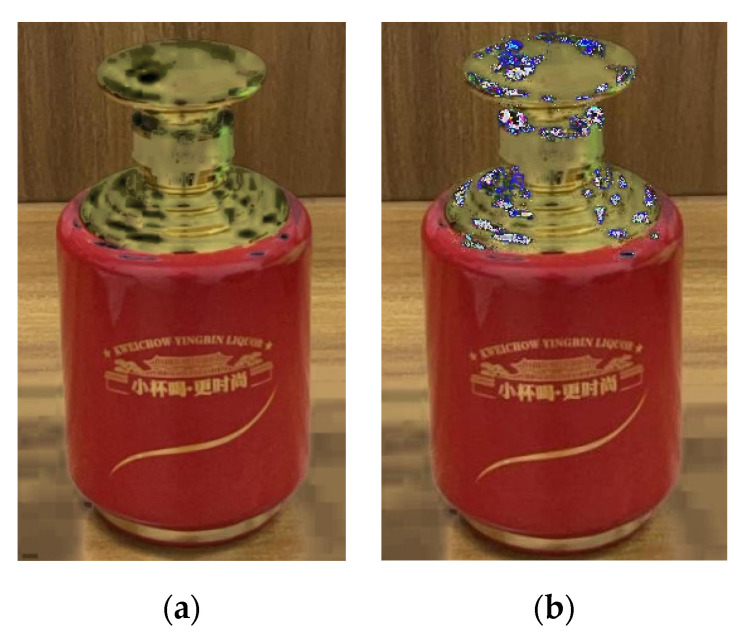
Sun et al.’s processing results of multiregion complex highlights on smooth liquor bottles: (**a**) specular reflection component separation results; (**b**) the result of the area filling method processing on the basis of the former.

Based on this idea, this paper adopts the same method to remove the highlight of the experimental objects of the self-made dataset. Figure 5 shows a liquor bottle with the surface highlight phenomenon treated by this method, and Figure 5a shows the specular component separation result. Specular separation theory was used to subtract the specular component from the pixel to obtain the diffuse component; because the specular reflection component was too large, the pixel information of the highlight area was destroyed after separation, and the color features of the original image could not be retained. Figure 5b shows the results of the region filling method based on the former. Surprisingly, the same method seemed to be ineffective on our experimental subjects. The reason is that the number of highlight areas to be filled on the surface of the liquor bottle was relatively large and the surface texture was complex. The traditional area filling repair algorithm was slow in this case and the visual effect was general.

**Figure 6:**
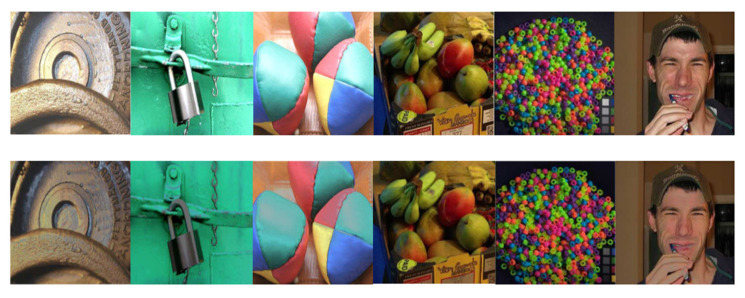
Experimental results of sparse and low-rank reflection model on Guo et al.’s dataset (the first row is the input highlight image; the second row is highlight processing map).

**Figure 7:**
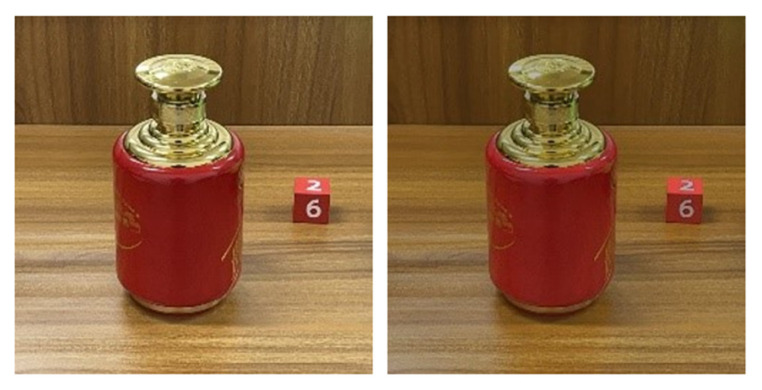
Experimental results of the same method on self-made datasets (the left side is the input highlight image; the right side is the highlight processing image).

Different from Antonio C.S. Souza [21], Guo et al. [15] proposed another optimization method based on the two-color reflection model. The principle of the method is based on the principle of the two-color reflection model. Different but interestingly, the method proposes a sparse and low-rank reflection model based on it. The inspiration for the proposed model comes from the distribution characteristics of the highlight phenomenon on the natural image, that is, the highlight of the natural image is usually strong but the distribution of the highlight is quite sparse, and the softer diffuse reflection on the image can be well approximated by a linear combination of several sparse different colors and low-rank weighting matrices. Through this reflection model, the highlight removal task was further transformed into a constrained nuclear norm and l1 norm minimization problem, which was finally solved by the Augmented Lagrange multiplier method. Figure 6 shows the effect of Guo et al. using this method on their experimental data. Based on their dataset, it can be seen that the highlight represented on each image was removed to some extent. Although the overall brightness of the image decreased, the processing effect for the highlight area performed well overall. Figure 7 shows the processing effect of the same method on our dataset. Compared with the characteristics of the highlight phenomenon in the original image of Figure 6, the highlight on the surface of the liquor bottle was more intense, complex and dense, resulting in the final highlight removal effect being not obvious, only the change in the overall brightness of the image.

**Figure 8:**
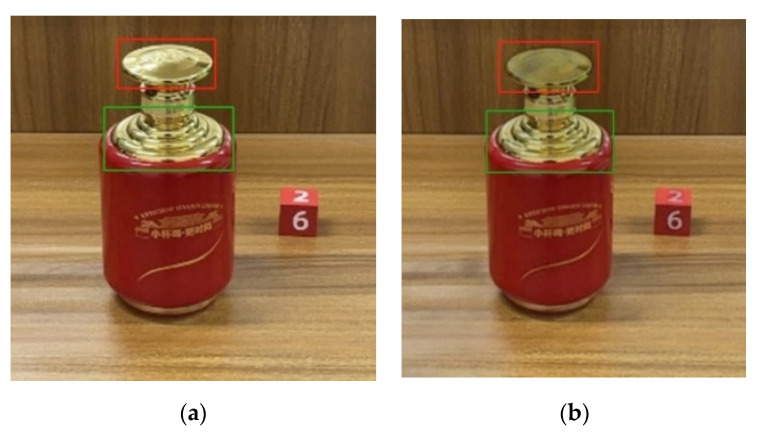
Test results of a multitask network model for joint highlight detection and removal: (**a**) input image; (**b**) output image.

Compared with traditional methods, the method based on deep learning greatly makes up for the shortcomings of traditional methods in image semantic understanding and solves the semantic gap between the low-level features and high-level semantics of images to a certain extent. Among them, the more advanced and representative is a novel joint highlight detection and removal multitask network proposed by Fu et al., which aims to detect and remove bright spots in natural images and achieved excellent results on their dataset. Unfortunately, they only published the test model code, and the test model is trained based on the SHIQ dataset. In the face of highly targeted highlight images, the network cannot perform well, so it is not suitable for our own research objects. Figure 8 shows the test results of the multitask network model for joint highlight detection and removal. It can be seen that only the highlights in the red box area were removed while the highlights in the green box area were still retained due to the complicated highlight phenomenon and texture.

Therefore, due to the limitations of the existing advanced traditional method and some methods based on deep learning in the highlight removal principle, network training and model parameter adjustment, these methods were not effective in the face of some practical problems and cannot be well applied.

## 3. Proposed Method

As shown in Figure 5, the priority region filling algorithm often fails to produce a satisfactory performance when facing objects with a large number of areas to be filled and complex surface textures. The reason is that the traditional image inpainting method is mainly based on content similarity and the correlation between image pixels to speculate repair. However, the computer itself does not have the same understanding and perception of images as human beings, resulting in problems such as blurred content and lack of semantics in the image restoration of complex missing areas. In recent years, deep learning technology has made a qualitative leap and has made a series of outstanding achievements in many research fields. Among them, the image inpainting method based on deep learning has also achieved remarkable results, which greatly makes up for the shortcomings of traditional methods in image semantic understanding and solves the semantic gap between the low-level features and high-level semantics of images to a certain extent. LaMa, a new image inpainting network proposed last year, has improved the latest technology for a range of datasets to achieve excellent performance even in challenging scenarios. On the basis of the trained BigLaMa model, we only need to input the paired original images and mask images into the network to easily remove the surface highlight. At the same time, since the LaMa network needs to input paired original images and mask images, and manually marking the mask images one by one is time consuming and laborious, this paper combines the U^2^-Net network before inputting the highlight image into LaMa to more efficiently exert the image inpainting performance of LaMa. The trained U^2^-Net highlight detection model can automatically and quickly detect the highlight on the surface of the bottle in large quantities and generate a mask map corresponding to the highlight area so that the two networks can work together to remove the highlight on the surface of the bottle.

**Figure 9:**
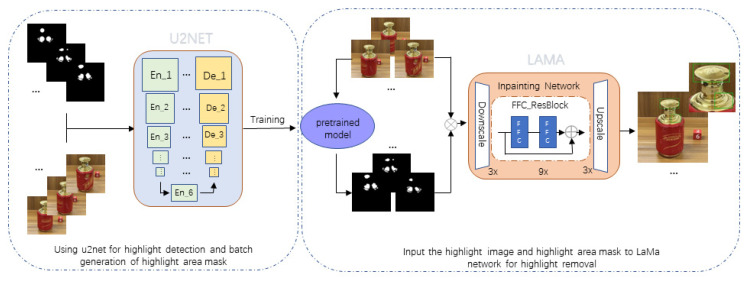
Flowchart of highlight processing combining U^2^-Net and LaMa networks.

A brief flow chart of our proposed method is shown in Figure 9.

In order to more effectively remove the highlight phenomenon on the surface of smooth liquor bottles, this paper is dominated by LaMa, and the removal of highlight is divided into two stages, whereby each stage has its core task. In the first stage, based on the trained U^2^-Net detection model, it is used to detect the specular reflection component in the input bottle image and generate a mask map for the highlight area in batches. In the second stage, the input image of the liquor bottle and the mask image generated by U^2^-Net are input into the LaMa network, and the removal of the highlight on the surface of the smooth liquor bottle is completed by the powerful image inpainting performance of LaMa.

### 3.1. The First Stage Task

Specifically, in the first stage, we focused on inputting the bottle image with a surface highlight and the mask image generated by manual labeling for the highlight area into the U^2^-Net network [19] for training, driving the network to learn the accurate extraction of the highlight area features, and finally obtaining the highlight detection model so that it can automatically detect the highlight on the surface of the bottle and generate the mask image for the highlight area in batches.

The strong highlight on the liquor bottle surface can be detected as the most attractive object in the segmented image according to its own saliency characteristics, and it can be regarded as a salient object for detection to some extent. Most of the existing SOD networks focus on making full use of the deep features extracted from the existing backbone networks. For example, Alexnet [23], VGG [24], ResNet [25], ResNeXt [26] and so on all use small convolutional filters with a size of 1 × 1 or 3 × 3 as feature extraction components. Such convolutional filters require less storage space and have a high computational efficiency, but the disadvantage is that their receptive fields are small. They focus on using the existing backbone network to extract deep features, and the extracted features are used to represent semantic information rather than local details and global comparison information. However, for bottle surface highlight detection, both local details and global context information are important.

In order to obtain more global information in the shallow feature map, the first thought is to expand the receptive field. Therefore, the RSU blocks used in U^2^-Net are proposed to capture multiscale features. The RSU structure integrates receptive field features of different scales, enabling the network to capture more contextual information, and the pooling operation in it ensures that the computational cost will not be significantly increased when increasing the overall architecture depth.

**Figure 10:**
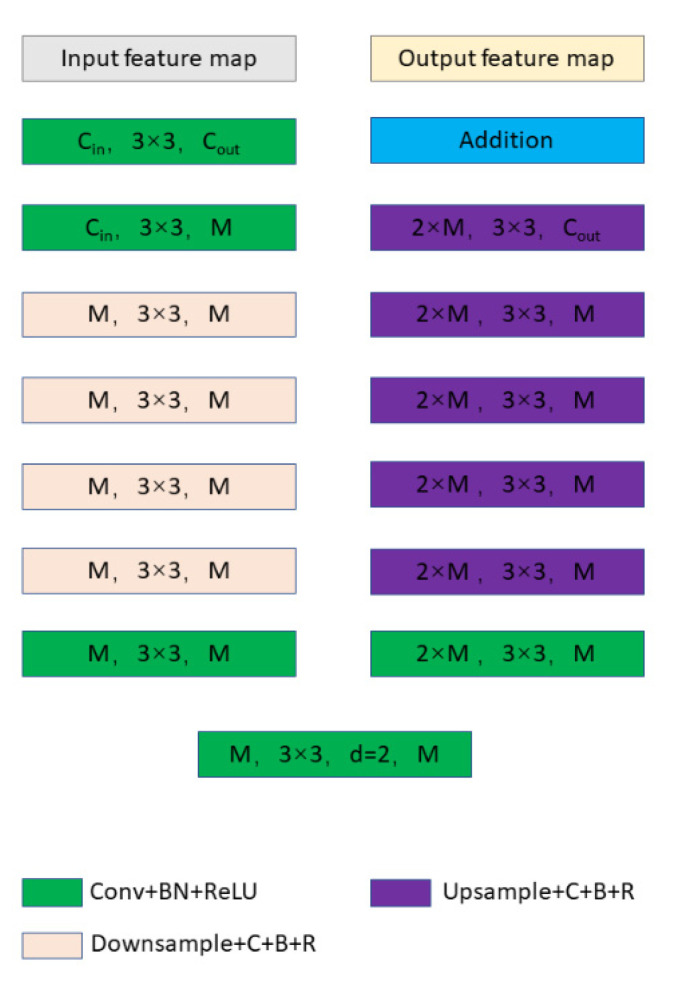
RSU-L.

The residual U block structure is shown in Figure 10, where M represents the number of channels in the inner layer of the RSU. This architecture enables us to restart the training network to meet our needs for highlight detection on the surface of the liquor bottle. RSU consists of three parts:

1. The input convolution layer, an ordinary convolution layer for local feature extraction, converts the input feature map $X\left(H,\times ,W,\times ,C_{in}\right)$ into an intermediate feature map $F_{1}\left(X\right)$ with $C_{out}$ channels.
2. Similar to U-Net’s symmetric encoder–decoder structure with a height of L, it takes the intermediate feature map $F_{1}\left(X\right)$ as the input and learns to extract and encode multiscale context information $U\left(F_{1}\left(X\right)\right)$. The depth of the residual U block (RSU) varies with the size of L, and a larger L means more pooling operations, a wider receptive field and richer local and global features.
3. Residual connections that fuse local and multiscale features: $F_{1}\left(X\right)+U\left(F_{1}\left(X\right)\right)$.

In terms of the loss function, the training loss is defined as:

(1) $$L=\sum_{m=1}^{M}w_{\mathrm{side}\;}^{\left(m\right)}{𝓁}_{\mathrm{side}\;}^{\left(m\right)}+w_{\mathrm{fuse}\;}{𝓁}_{\mathrm{fuse}},$$

where ${𝓁}_{\mathrm{side}}^{\left(m\right)}$ is the loss of the output saliency map $S_{\mathrm{side}}^{\left(m\right)}$ and ${𝓁}_{\mathrm{fuse}}$ is the loss of the final fusion output saliency map. $w_{\mathrm{side}}^{\left(m\right)}$ and $w_{\mathrm{fuse}}$ represent the weight of each loss. Meanwhile, standard binary cross-entropy is used to calculate the loss size:

(2) $$𝓁=-\sum_{\left(r,c\right)}^{\left(H,W\right)} [P_{G\left(r,c\right)}\log P_{S\left(r,c\right)}+\left(1-P_{G\left(r,c\right)}\right)\log\left(1-P_{S\left(r,c\right)}\right)],$$

Among them, $\left(r,c\right)$ represents pixel coordinates; $\left(H,W\right)$ represents image resolution; and $P_{G\left(r,c\right)}$ and $P_{S\left(r,c\right)}$ represent the GT pixel values and predicted saliency probability maps, respectively. The final fusion result $l_{\mathrm{fuse}}$ is selected as the final saliency map.

### 3.2. The Second Stage Task

This stage takes LaMa as the core. On the basis of the U^2^-Net highlight detection model that has been trained, the highlight image of the liquor bottle surface is input into the U^2^-Net network for highlight detection, and the mask map corresponding to the highlight area is automatically generated in batches. Then, the mask image and the bottle highlight image are input to the LaMa network at the same time, and the surface highlight of the smooth liquor bottle is removed by relying on the powerful repair performance of the LaMa network.

The inspiration of using LaMa [20] to remove the highlight on the surface of the liquor bottle comes from the deficiency of the traditional image inpainting method in principle. It mainly infers the inpainting based on content similarity and the correlation between the image pixels. However, the computer itself does not have the same understanding and perception of images as human beings, resulting in problems such as blurred content and a lack of semantics in the image restoration of complex missing areas; Figure 5b reflects this problem. The image inpainting method based on deep learning greatly makes up for the shortcomings of traditional methods in image semantic understanding. This method pays more attention to ‘understanding‘ the overall scale structure of natural images, and on this basis, the missing parts are synthesized to solve the semantic gap between the low-level features and high-level semantics of images. Thus, the highlight on the surface of the bottle can be regarded as the missing area to be repaired to a certain extent, and we can make full use of the ability of the repair network to capture the high-level semantic information of the picture to accurately and effectively remove the highlight.

In this paper, the BigLaMa model, which has been trained by the author of the original LaMa paper, is used to remove the highlight area on the surface of the liquor bottle. Compared with the standard LaMa model, BigLaMa has a deeper generator depth and is based on a larger training dataset and batch size. It was trained on the Places Challenge dataset [27], which has approximately 4.5 million images and has been trained on eight NVidia V100 GPUs for approximately 240 h, making it more applicable.

Typically, the training of an image inpainting network is based on real images and large image–mask pair datasets created by randomly masking portions of the real image. Therefore, during training, a large effective receptive field [28] is essential to understand the global structure of the image and thus solve the repair problem. In addition, even if the mask is large but the receptive field is insufficient, it will still lead to a poor repair effect. Therefore, a good network architecture should have the widest possible receptive field units in the channel as early as possible. Traditional fully convolutional models, such as ResNet, usually have small convolution kernels (e.g., 3 × 3), resulting in insufficient receptive fields in some cases, especially in the early layers of the network, and in many layers in the network lacking global context information.

**Figure 11:**
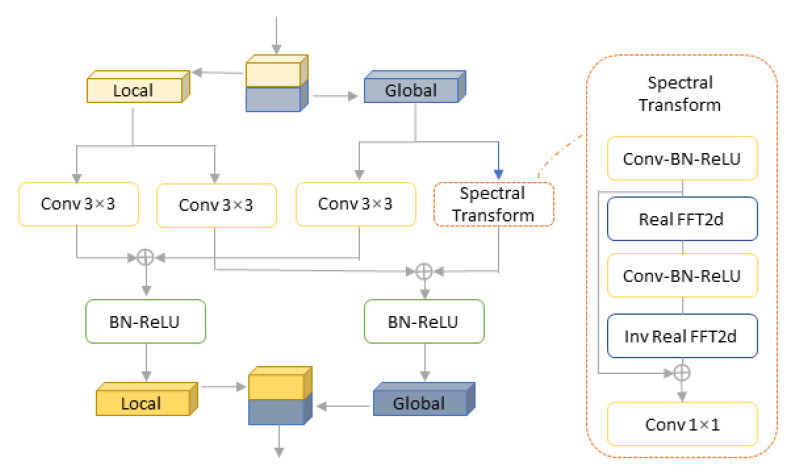
The fast Fourier convolution model.

Most of the existing image inpainting systems lack effective receptive fields to a certain extent due to the influence of their own inpainting networks and loss functions, resulting in a significant decline in inpainting performance when facing complex geometric structures and high-resolution images in natural scenes. In order to alleviate the impact of this problem, a method called large mask repair (LaMa) was born. Usually, the key premise to effectively solve the problem of image inpainting is to have a large effective receptive field, which is very important for understanding the global structure of the image and solving the inpainting problem. Therefore, in view of the problem that the commonly used popular convolutional architectures may lack sufficient effective receptive fields, the advantages of LaMa stand out. Specifically, its advantages are mainly reflected in the following aspects:

1. The fast Fourier convolution model (FFC) [29] instead of the traditional full convolution model greatly improves the efficiency of convolution operation and allows the network to consider the global context information at an earlier stage, covering the receptive field of the whole image. Figure 11 shows the fast Fourier convolution model.
2. The perceptual loss [30] based on the semantic segmentation network with a high receptive field is adopted to promote the consistency of the global shape structure. Compared to the original supervised loss, the perceptual loss does not require the generator to accurately reconstruct the ground truth, which evaluates the distance between the extracted features in the target image and the image through a basic pretrained network, making LaMa repairs focus on understanding the global structure.
3. A more effective mask generation strategy is adopted in the training process to generate wide and large masks so that the network can fully utilize the high receptive field of the model and the performance of the loss function.

In terms of the loss function, as described above, the high receptive field perceptual loss $\left(\mathrm{HRFPL}\right)$ uses the high receptive field basic model $\phi_{HRF}\left(\cdot \right)$:

(3) $$L_{HRFPL}\left(x,\hat{x}\right)=M\left({ [\phi_{HRF}\left(x\right)-\phi_{HRF}\left(\hat{x}\right)]}^{2}\right)$$

where M stands for sequential two-stage mean operation, $\phi_{HRF}\left(x\right)$ is implemented by using Fourier or dilated convolution, and [· − ·]^2^ stands for pixel-wise operation.

On this basis, an adversarial loss is chosen to ensure that the local details of the appearance of the images generated by the inpainting model $f_{\theta }\left(x^{\prime }\right)$ are natural and realistic. $D_{\xi }\left(x\right)$ is a defined discriminator (used to distinguish between ‘true‘ and ‘false‘ patches; patches that intersect with the mask area are marked with a ‘false‘ label, and known parts of the generated image are marked with a ‘true‘ label). At the same time, a nonsaturating adversarial loss is used:

(4) $$\begin{array}{cc} L_{D}=-E_{x}[ & \log D_{\xi }\left(x\right)]-E_{x,m} [\log D_{\xi }\left(\hat{x}\right)⊙m]\\ & -E_{x,m} [\log\left(1-D_{\xi }\left(\hat{x}\right)\right)⊙\left(1-m\right)] \end{array}$$

(5) $$L_{G}=-E_{x,m} [\log D_{\xi }\left(\hat{x}\right)],$$

(6) $$L_{Adv}={\mathrm{sg}}_{\theta }\left(L_{D}\right)+\mathrm{sg}g_{\xi }\left(L_{G}\right)\to \underset{\theta ,\xi }{min},$$

where x is the sample from the dataset, M is the randomly generated mask according to the large mask policy, $\hat{x}=f_{\theta }\left(x^{\prime }\right)$ is the inpainting result for $x^{\prime }=\mathrm{stack}\left(x⊙m,m\right)$ and $L_{Adv}$ is the joint loss to be optimized.

It is well known that $L_{\mathrm{DiscPL}\;}$ can make the training process more stable and improve the performance of the network to a certain extent. Thus, the gradient penalty $R_{1}=E_{x}∥\nabla D_{\xi }\left(x\right){∥}^{2}$ [31,32] and feature matching loss $L_{\mathrm{DiscPL}}$ are also used in the final loss, and the final loss function of the LaMa inpainting network is:

(7) $$L_{\mathrm{final}\;}=\kappa L_{\mathrm{Adv}\;}+\alpha L_{\mathrm{HRFPL}\;}+\beta L_{\mathrm{DiscPL}\;}+\gamma R_{1}.$$

where $L_{\mathrm{Adv}}$ and $L_{\mathrm{DiscPL}}$ are responsible for generating natural and realistic local details of the image, while $L_{\mathrm{HRFPL}}$ is responsible for the consistency of the global structure and supervision signals.

## 4. Results

In this section, we first introduce the source of the experimental data and the corresponding evaluation criteria and elaborate the experimental details. Then, we show the experimental results of this method and compare the final highlight removal results with some other advanced methods to verify the advantages and effectiveness of our method.

### 4.1. Datasets and Evaluation Metric

This paper verifies the effectiveness of the method by using a self-made liquor bottle surface highlight dataset. The dataset is a small sample dataset, which contains about 200 real images of each angle in the horizontal direction in the same scene. More importantly, the sample data has the characteristics of a smooth surface material, strong reflection and dense highlight area. Additionally, all the original data were collected in the industrial simulation environment, and each image has different degrees of surface highlights. For evaluation, in order to comprehensively evaluate the detection and removal effect of the highlight on the surface of the liquor bottle by our proposed method, in addition to the visual evaluation, we also selected the following metrics as indicators for performance evaluation: accuracy; recall rate; F-measure; peak signal noise ratio (PSNR); and structural similarity (SSIM). Among them, the PR curve, MAE and F-measure are mainly used to evaluate the detection performance of U^2^-Net for highlights on the surface of liquor bottles. PSNR and SSIM [33] are used to evaluate the quality of the whole image after highlight removal.

### 4.2. Implementation Detail

The method proposed in this paper was implemented in Pytorch and was based on U^2^-Net and LaMa networks. The training and testing processes were carried out on the Linux Ubuntu 20.04 operating system equipped with a CPU of i5-12400F and a GPU of NVIDIA GeForce RTX 3060 (12GB memory).

For U^2^-Net, since no existing backbone is used in the network, we trained the network model from scratch. During the training process, the size of the 200 bottle images was first adjusted to 320 × 320 and then randomly flipped vertically and cut to 288 × 288. All convolutional layers were initialized by Xavier, and the loss weights $w_{\mathrm{side}}^{\left(m\right)}$ and $w_{\mathrm{fuse}}$ were set to one. We used the Adam optimizer to train our network, and the hyperparameters were set to the default values (initial learning rate = 1 × 10^−3^, betas = (0.9, 0.999), eps = 1 × 10^−8^, weight decay = 0). We trained the network until the loss converged (after about 1500 iterations), and we did not use the verification set part of the previous method [34]. The entire training process took about 6 h. During the test, the size of the input image was adjusted to 320 × 320 and was input into the network to obtain the predicted saliency map. The predicted saliency map of 320 × 320 was adjusted back to the same size as the input image, and the bilinear interpolation method was used in the adjustment process.

For LaMa, we used the Big LaMa model, which has been trained by Roman Suvorov et al. Big LaMa-Fourier is different from the standard model LaMa-Fourier in three aspects:

1. The depth of the generator varies, with a total of 18 FFC-based residual blocks.
2. Different training datasets; the Big LaMa model was trained on approximately 4.5 million images in the Places Challenge dataset [27].
3. The batch sizes are different, with Big LaMa using a larger batchsize of 120. It has been trained on 8 NVidia V100 GPUs for about 240 h.

### 4.3. Comparison with Other Methods

Based on the self-made dataset, we compared the proposed method with some advanced and representative methods to demonstrate our advantages.

**Figure 12:**
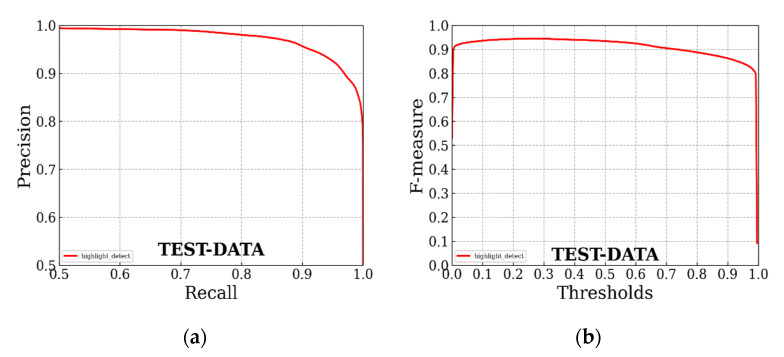
PR curve and comprehensive evaluation index curve of U^2^-Net model on self-made dataset: (**a**) PR curve; (**b**) comprehensive evaluation index curve.

**Figure 13:**
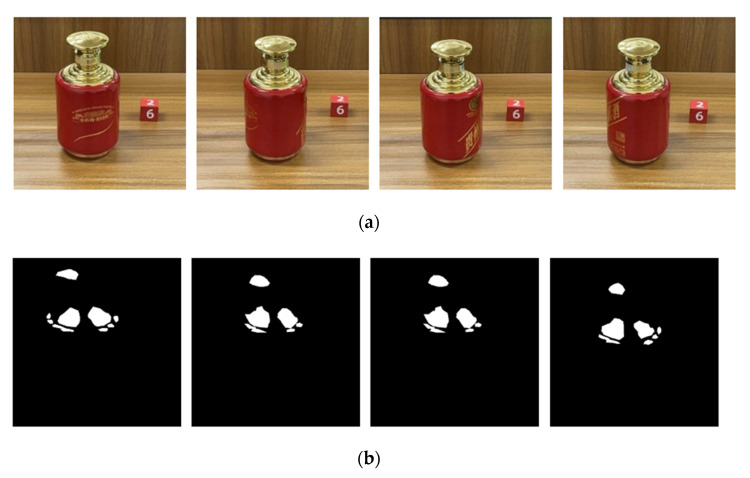

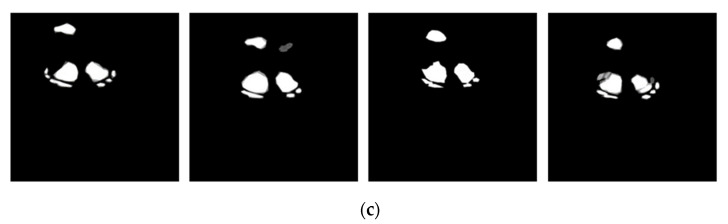
Partial test results of our U^2^-Net on real surface highlight bottle images: (**a**) original input image; (**b**) ground Truth; (**c**) result map.

The PR curve and comprehensive evaluation index curve (F-measure) of our highlight detection model on the self-made dataset are shown in Figure 12. It shows the excellent performance of our U^2^-Net on the self-made dataset, and the actual highlight detection results of the model met the needs of subsequent highlight removal. In addition, an example of the partial detection results of our U^2^-Net specular detection model on real surface highlight liquor bottle images is shown in Figure 13.

**Figure 14:**
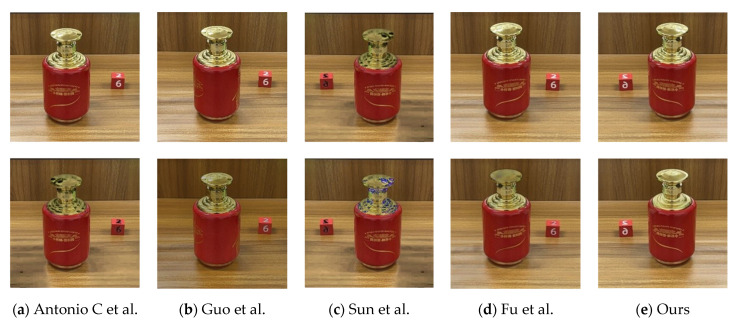
Visual comparison of our proposed highlight removal method with other state-of-the-art methods (the first row is the input image, and the second row is the image processed by the respective algorithms): (**a**) Antonio C et al. test result image; (**b**) Guo et al. test result image; (**c**) Sun et al. test result image; (**d**) Fu et al. test result image; (**e**) our test result image.

**Visual comparison.**Figure 14 visually compares the highlight removal results generated by our method with other state-of-the-art highlight removal methods. Specifically, traditional methods based on two-color reflection models [15,21], as shown in Figure 14a,b, often incorrectly detect white textures in images as highlight or fail to recognize highlight because they cannot semantically distinguish between highlight areas and white material surfaces. In addition, the priority region filling theory [22] shown in Figure 14c cannot be well applied in the face of the multiregion complex highlight phenomenon in the image. The method based on deep learning [18] shown in Figure 14d achieved better highlight detection and removal effects than other highlight removal algorithms in three large datasets (SHIQ, CLH and LIME). Unfortunately, they only published the test model code, and the test model was trained on the SHIQ dataset. In the face of highly targeted highlight images, the network cannot perform well, so it is not suitable for our own research objects, and it failed to achieve the expected results on our liquor bottle highlight dataset. In contrast, our method shown in Figure 14e can detect and remove highlight more accurately, and our results are more consistent with the basic facts.

**Quantitative comparison.** Table 1 reports the PSNR and SSIM values of different methods based on self-made datasets, which shows that our proposed method had higher PSNR and SSIM scores than other compared methods.

It can be seen from Table 1 that the proposed method achieved very good results in both PSNR and SSIM scores, and SSIM achieved the best results. Although Sun’s method achieved the second best results, the surface of the highlight image processed by this method had a large area of content blur and semantic loss, which is inconsistent with the objective facts.

## 5. Conclusions

In this paper, we propose a fast specular highlight removal method for a smooth liquor bottle surface combined with a U^2^-Net and LaMa model based on a new highlight removal idea. The method is simple, effective and easy to implement. By accurately exerting the respective saliency detection and image inpainting performances of U^2^-Net and LaMa, the highlight on the bottle surface can be efficiently located and removed. At the same time, the effectiveness of this kind of joint network for some highly targeted small sample highlight datasets is also well explored. A large number of experimental verifications showed that based on the self-made small sample liquor bottle surface highlight dataset, this method shows better results than other advanced algorithms in both visual and image quality indicators.

## Figures and Tables

**Table 1:** Quantitative comparison of the proposed method with other advanced highlight removal methods.

Methods	PSNR	SSIM
Antonio C. et al. [21]	18.621	0.843
Guo et al. [15]	18.291	0.879
Sun et al. [22]	26.969	0.972
Fu et al. [18]	24.837	0.880
Ours	29.873	0.966

## Data Availability

The datasets used in the current study are available from the corresponding author upon reasonable request.
